# Effects of El Niño Southern Oscillation and Dipole Mode Index on Chikungunya Infection in Indonesia

**DOI:** 10.3390/tropicalmed5030119

**Published:** 2020-07-16

**Authors:** Harapan Harapan, Amanda Yufika, Samsul Anwar, Haypheng Te, Hamzah Hasyim, Roy Nusa, Pandji Wibawa Dhewantara, Mudatsir Mudatsir

**Affiliations:** 1 Medical Research Unit, School of Medicine, Universitas Syiah Kuala, Banda Aceh, Aceh 23111, Indonesia; amandayufika@gmail.com (A.Y.); mudatsir@unsyiah.ac.id (M.M.); 2 Department of Microbiology, School of Medicine, Universitas Syiah Kuala, Banda Aceh, Aceh 23111, Indonesia; 3 Tropical Disease Centre, School of Medicine, Universitas Syiah Kuala, Banda Aceh, Aceh 23111, Indonesia; 4 Department of Family Medicine, School of Medicine, Universitas Syiah Kuala, Banda Aceh, Aceh 23111, Indonesia; 5 Department of Statistics, Faculty of Mathematics and Natural Sciences, Universitas Syiah Kuala, Banda Aceh, Aceh 23111, Indonesia; samsul.anwar@unsyiah.ac.id; 6 Siem Reap Provincial Health Department, Ministry of Health, Siem Reap 1710, Cambodia; hayphengte@gmail.com; 7 Faculty of Public Health, Sriwijaya University, Indralaya, South Sumatra 30862, Indonesia; hamzah@fkm.unsri.ac.id; 8 Vector-Borne Disease Control, Research and Development Council, Ministry of Health, Jakarta 10560, Indonesia; roynres@gmail.com; 9 Pangandaran Unit of Health Research and Development, National Institute of Health Research and Development (NIHRD), Ministry of Health of Indonesia, West Java 46396, Indonesia; p.dhewantara@uq.edu.au; 10 UQ Spatial Epidemiology Laboratory, School of Veterinary Science, The University of Queensland, Gatton, QLD 4343, Australia

**Keywords:** chikungunya, Dipole Mode Index, El Niño, ENSO, Indian Ocean Dipole

## Abstract

The aim of this study was to assess the possible association of El Niño Southern Oscillation (ENSO) and Dipole Mode Index (DMI) on chikungunya incidence overtime, including the significant reduction in cases that was observed in 2017 in Indonesia. Monthly nation-wide chikungunya case reports were obtained from the Indonesian National Disease Surveillance database, and incidence rates (IR) and case fatality rate (CFR) were calculated. Monthly data of Niño3.4 (indicator used to represent the ENSO) and DMI between 2011 and 2017 were also collected. Correlations between monthly IR and CFR and Niño3.4 and DMI were assessed using Spearman’s rank correlation. We found that chikungunya case reports declined from 1972 cases in 2016 to 126 cases in 2017, a 92.6% reduction; the IR reduced from 0.67 to 0.05 cases per 100,000 population. No deaths associated with chikungunya have been recorded since its re-emergence in Indonesia in 2001. There was no significant correlation between monthly Niño3.4 and chikungunya incidence with *r* = −0.142 (95%CI: −0.320–0.046), *p* = 0.198. However, there was a significant negative correlation between monthly DMI and chikungunya incidence, *r* = −0.404 (95%CI: −0.229–−0.554) with *p* < 0.001. In conclusion, our initial data suggests that the climate variable, DMI but not Niño3.4, is likely associated with changes in chikungunya incidence. Therefore, further analysis with a higher resolution of data, using the cross-wavelet coherence approach, may provide more robust evidence.

## 1. Background

Chikungunya, caused by chikungunya virus (CHIKV), is one of the most epidemiologically important infectious diseases globally, together with dengue and Zika [1,2]. CHIKV is a small, spherical, and enveloped virus and belongs to the *Alphavirus* genus [3]. An outbreak of chikungunya was first reported in Tanzania in 1952 [4,5], with sporadic outbreaks subsequently identified in Africa and Asia during the 1950s and 1960s [6]. CHIKV re-emerged in the early of 2000s and caused massive outbreaks in several islands of the Indian Ocean and Southeast Asia [7]. In Réunion Island, a French department in the Indian Ocean, chikungunya affected about a third of the population [8,9], while in India the virus infected more than 1.3 million persons during 2005−2006 [10]. A contemporary systematic review indicates that more than 20 countries in the Asia-Pacific had reported chikungunya outbreaks since its modern re-emergence [11].

Chikungunya is endemic in Indonesia [12]. Historical report suggests that CHIKV has circulated in the capital, Jakarta, since 1779 [13,14]. Serological studies between 1969–1972 also demonstrated significant titters of anti-CHIKV antibodies in most of the Indonesian archipelago when tested using hemagglutination inhibition assay and the plaque reduction neutralization test [15,16]. However, the first official chikungunya cases recorded by the Indonesian Ministry of Health (MoH) occurred in Samarinda of Kalimantan Island in 1973 [17]. The first virologically confirmed chikungunya outbreak was reported in Jambi (Sumatra Island) in 1982 [18]. Multiple outbreaks were reported between 1983 and 1984 before a 20-year period where disease cases were not reported in Indonesia [12]. Chikungunya re-emerged in Indonesia in 2001, and has since caused multiple outbreaks in the country [17]. The highest number of cases of chikungunya was recorded during 2009−2010, where 137,655 cases were reported [12].

In 2017, a significant decline of chikungunya cases was observed where the cases reduced from 1972 cases in 2016 to just 126, nation-wide. Interestingly, in the same year, a sharp reduction of dengue, a disease caused by a virus that shares similar transmission characteristics and ecological features to CHIKV, was also noticed in Indonesia [12] as well as in several countries in the Americas [19]. Some hypotheses have been proposed for the reduction of the dengue, including changes in the density and competencies of vectors, due to changes in climatic conditions, cross-immunity generated by the simultaneous circulation of several arboviruses, and changes in epidemiological surveillance systems [19]. A study was conducted to determine the factors associated with the reduction of dengue in 2017 in Indonesia [12], but no such study exists for chikungunya.

Previous studies have hypothesized a potential association between climate variability and chikungunya infection [20,21]. Two of the most important indicators used for climate changes are El Niño Southern Oscillation (ENSO) and Indian Ocean Diplo (IOD). These indicators link with a dry and warm season that potentially increase mosquito vector breeding and shorter virus extrinsic incubation periods [21]. The role of these indicators in other infections such as dengue, malaria, hantavirus, Rift Valley fever, cholera, and Zika also have been studied, with variable results [21,22,23,24,25,26,27]. The aims of this study are (a) to investigate the reduction of chikungunya cases that occurred in Indonesian in 2017; and (b) to assess the effects of ENSO and DMI on chikungunya incidence in Indonesia, overtime, since its re-emergence.

## 2. Materials and Methods

### 2.1. Variables and Data Sources

#### 2.1.1. Chikungunya Notifications

National chikungunya case reports were obtained from National Disease Surveillance, the Directorate General of Disease Prevention and Control, MoH of Health of Indonesia. The chikungunya case definition used in this surveillance system has been published in the National Guideline for Prevention and Control of Chikungunya [28], which follows the World Health Organization criteria [29]. In brief, chikungunya cases were classified into three categories: (a) possible case, diagnosed based on clinical criteria alone, including acute onset of fever (>38.5 °C) and severe arthralgia/arthritis that is not explained by other medical conditions; (b) probable case, diagnosed based on the clinical criteria as mentioned and epidemiological criteria (residing or having visited epidemic areas) and; (c) confirmed case, diagnosed based on laboratory criteria which show a positive result for virus isolation, reverse transcription-polymerase chain reaction (RT-PCR), IgM antibodies, or the demonstration of a four-fold increase in IgG antibodies [12]. The surveillance system includes chikungunya cases as defined by each of these three categories. In this study, monthly and annual time series of chikungunya cases between 2001 and 2017 were used.

#### 2.1.2. El Niño Southern Oscillation (ENSO)

ESNO is periodic fluctuation in sea surface temperature (SST) and the air pressure of the overlying atmosphere across the equatorial Pacific Ocean. It is one of the most important modes of variability impacting the climate in the tropics and subtropics [30,31]. ENSO primarily focuses on SST anomalies in four geographic regions of the equatorial Pacific Ocean. In this study, Niño3.4 was used to represent the ENSO. Niño3.4, comprising a portion of Niño regions 3 and 4, is the average SST anomaly in the region bounded by 5° N to 5° S, and from 170° W to 120° W. Niño3.4 data were obtained from the Earth System Research Laboratory of the National Oceanic and Atmospheric Administration (NOAA) [32]. Monthly Niño3.4 data, collected between 2011 and 2017 were used for analysis.

#### 2.1.3. Indian Ocean Dipole (IOD)

IOD is defined as the difference in SST between two poles: a western pole in the western Indian Ocean (located in the Arabian Sea, 50° E–70° E and 10° S–10° N) and an eastern pole in the eastern Indian Ocean south of Indonesia (90° E–110° E and 10° S–0° N) [33,34]. This gradient is called the Dipole Mode Index (DMI). Monthly average of DMI values were retrieved from Japan Agency for Marine-Earth Science and Technology (JAMSTEC) [35]. DMI values collected between 2011 and 2017 were analyzed in this study.

### 2.2. Data Analysis and Approaches

In 2001, after not recorded for approximately 20 years, CHIKV re-emerged in Indonesia. The annual incidence rate (IR) and annual case fatality rate (CFR) of chikungunya were calculated to characterize chikungunya activity in the country since its re-emergence (i.e., from 2001 and 2017). The national IR was calculated by dividing the total number of cases by the total Indonesian population at that time. The CFR is reported as the total number of deaths caused by chikungunya divided by the total number of cases reported in that same year. Annual IR and CFR were expressed as the number of cases per 100,000 population and as a percentage (%), respectively. The total Indonesian population was obtained from the Indonesian Bureau of Statistics. To determine the geographical distributions of chikungunya reduction in 2017, chikungunya IR and CFR of each province in 2016 and 2017 were calculated and mapped using ArcGIS software [36].

To assess the role of Niño3.4 and IOD and chikungunya cases in Indonesia, monthly time series data were used between 2011 and 2017. Correlations between the monthly Niño3.4 and DMI, and monthly IR and CFR values, were assessed using Spearman’s rank correlation. This analysis was used based on analysis of data normality by the Shapiro–Wilk test. For each correlation, 95% confidence interval (95% CI) was calculated and significance was assessed at α = 0.05.

## 3. Results

### 3.1. Chikungunya Incidence Rate Overtime and Its Reduction in 2017

**Figure 1:**
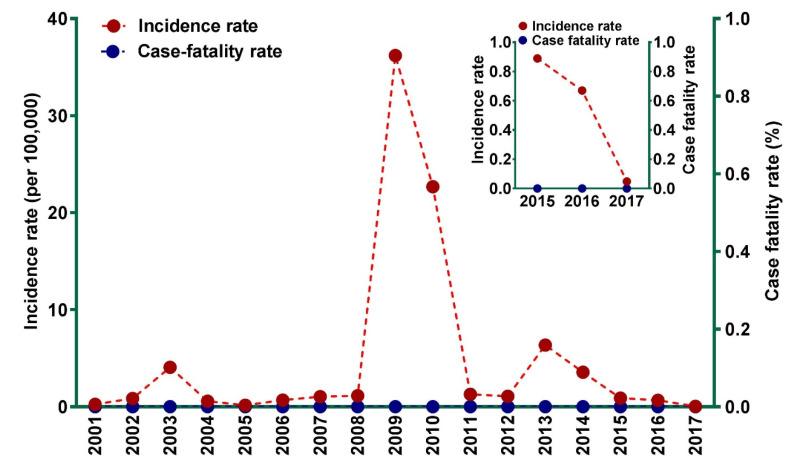
Incidence rate and case fatality rate of chikungunya between 2001 and 2017 in Indonesia. The insert shows details of reduction of chikungunya incidence rate in 2017.

Temporal distribution of chikungunya IR and CFR since its re-emergence in 2001 is presented in Figure 1. From 2001 to 2016, chikungunya IR ranged from 0.15 cases (in 2005) to 35.26 cases (in 2009) per 100,000 population. The highest number of infections during our study period was reported in 2009, with more than 83,000 cases. A large number of chikungunya cases were also reported in 2013 (Figure 1). No deaths associated with chikungunya were reported in Indonesia since its re-emergence in 2001.

The low number of chikungunya cases reported in 2017 is not exceptional in the context of the whole picture of chikungunya IR during our study period (2001 to 2017) (Figure 1). However, there was a substantial reduction of chikungunya cases in Indonesia from 2016 to 2017, with case reports declining from 1702 to 126 cases, corresponding to a 92.6% reduction. Chikungunya IR declined significantly from 0.67 (2016) to 0.05 (2017) cases per 100,000 population, a decline of 92.8% (Figure 1).

**Figure 2:**
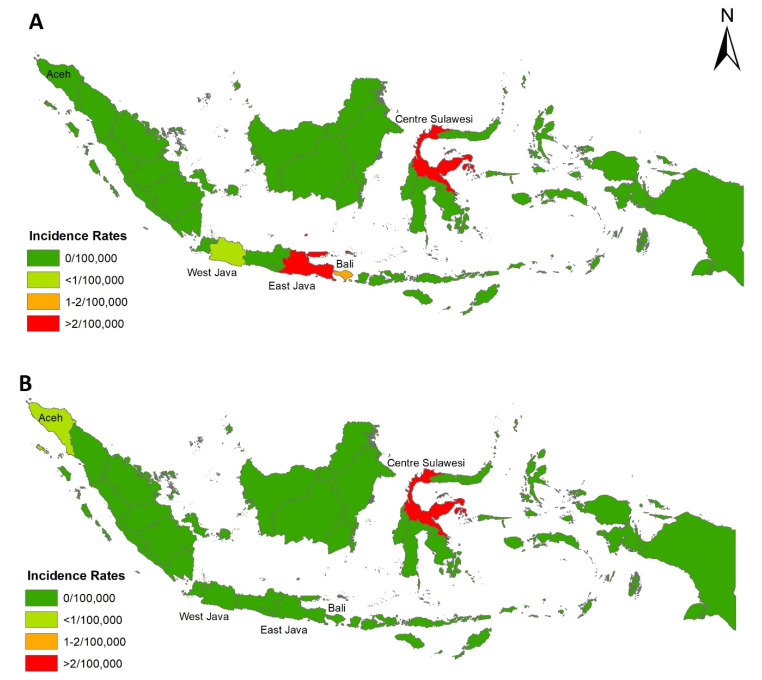
Provincial incidence rates of chikungunya in 2016 (**A**) and 2017 (**B**).

In 2016, chikungunya cases were reported in four provinces: East Java, Central Sulawesi, Bali, and West Java, with 1489, 103, 80, and 30 cases, respectively, accounting for IRs of 3.83, 3.58, 1.92, and 0.06 per 100,000 population. In 2017, however, chikungunya cases were reported in just two provinces: Central Sulawesi (121 cases) and Aceh (5 cases) with IRs of 4.07 and 0.09 per 100,000 population, respectively. Spatial distribution of chikungunya IR in 2016 and 2017 in each province are presented in Figure 2.

### 3.2. Correlation between Climate Variables and Chikungunya Incidence

#### 3.2.1. El Niño Southern Oscillation

**Figure 3:**
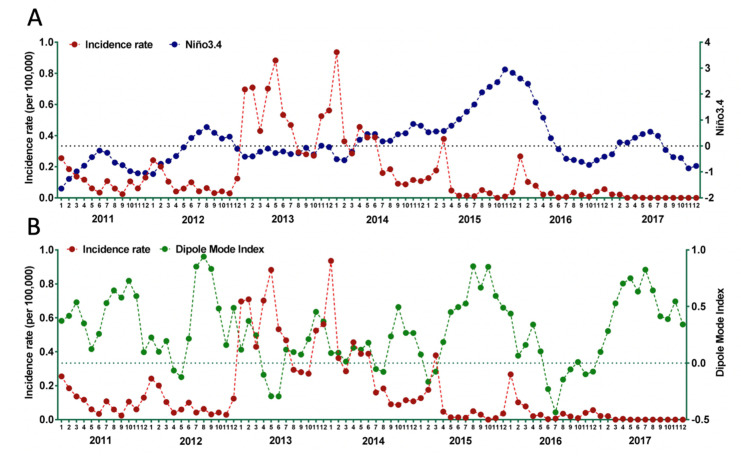
Temporal trend of Niño3.4 and chikungunya incidence rate (**A**) and DMI and chikungunya incidence rate (**B**) in Indonesia, 2005–2017.

Correlations between monthly Niño3.4 and chikungunya incidence for seven years (2011–2017) were calculated. The fluctuation of monthly Niño3.4 and chikungunya incidence are presented in Figure 3A. There was no significant correlation between Niño3.4 and chikungunya incidence with *r* = −0.142; 95%CI: −0.320–0.046, *p* = 0.198. However, data from 2014–2017 suggests that the rise of chikungunya incidence was influenced negatively by Niño3.4. With the exception of data from 2013, a negative correlation between Niño3.4 and chikungunya incidence rate was observed, where increase of Niño3.4 correlated with a decrease in chikungunya notifications.

#### 3.2.2. Indian Ocean Dipole

Correlations between monthly DMI and chikungunya incidence for seven years (2011–2017) was calculated. The fluctuation of monthly DMI and IR of chikungunya is presented in Figure 3B. There was a significant negative correlation between DMI and chikungunya incidence with *r* = −0.404; 95%CI: −0.229 – −0.554, *p* < 0.001. Decreased DMI (decreased difference in SST between western pole in the western Indian Ocean and eastern pole in the eastern Indian Ocean south of Indonesia), correlated with increased incidence of chikungunya. There was a clear association between increased incidence of chikungunya with negative DMI.

## 4. Discussion

### 4.1. Declined of Notified Chikungunya in 2017

A significant decrease of notified chikungunya cases in Indonesia was observed in 2017, compared to the previous year. The Indonesian MoH suggested that the reinforcement of vector eradication program named “one house one jumantik” (one house, one mosquito larvae’s monitor) contributed to the decline of dengue and chikungunya in that year [37]. However, a recent study indicated that the implementation of that program was not the main factor in reduction of dengue [12]. In 2017, chikungunya cases declined significantly to zero in three provinces (East Java, Bali, and West Java) but it did not occur in Central Sulawesi. Based on MoH data, only 15.8% and 28.9% of districts in East Java implemented integrated vector management, including a vector eradication program, in 2016 and 2017, respectively. Coverage was much higher in Central Sulawesi: 76.9% (2016) and 100% (2017). Chikungunya was also reported in Aceh in 2017, yet 100% of the districts in this province implemented the program. In some provinces such as Riau, West Sumatra, West Kalimantan, and Banten, no chikungunya cases were reported despite poor coverage of vector control programs, with <30% of districts covered in 2016 and 2017 [38,39]. This suggests that there are other factors contributing to the decline of chikungunya cases in Indonesia in 2017.

### 4.2. Climate Change Variables and Chikungunya Incidence

ENSO, the strongest interannual climate cycle, impacts on global climate and weather anomaly patterns with warm (El Niño) and cold phases (La Niña) of the cycle. ENSO is associated with drought and flood conditions, and one of the typical conditions of El Niño is dry conditions in Indonesia. Precipitation and temperature resulting from ENSO events are the background drivers of vector-borne disease activity [21,40,41,42]. The persistence of extreme conditions of temperature or precipitation impacts the ecology and habitat size, growth rates, dynamics, and distribution of the vector population, as well as viral replication and extrinsic incubation duration [20,21,43,44].

A recent study suggested severe ENSO-induced drought conditions in Southeast Asia were associated with increased water storage around houses and elevated ambient air temperatures. These factors contributed to elevated *Aedes* mosquito populations and reduced the extrinsic incubation period of CHIKV, respectively, and therefore contributed to the increase of chikungunya cases [21]. However, our findings suggest there is no significant correlation between Niño3.4 and chikungunya incidence. Some studies also found similar findings in the context of other infections. In the western Kenyan highlands, for example, no evidence of an association between Niño3 and the number of malaria cases was deduced [23]. There was a very weak association between ENSO and dengue incidence in Bangladesh [24]. However, in India, El Niño was positively correlated with dengue cases, with a 3–6 months lag period [25]. In Taiwan, temperature and precipitation influenced dengue incidence rates with a lag of 10–20 weeks [26]. In the context of dengue infection in Indonesia, studies have shown that dengue incidence is associated with temperature, rainfall, and humidity [45,46,47]. In this present study, those local climate variables (temperature, rainfall, and humidity), which might have had a stronger impact on chikungunya incidence, were not included. A previous study found that these local climate variables were more influential on dengue incidence than ENSO [24]. Another study found that ENSO had a weaker impact on the seasonality of local climate conditions than IOD [26].

IOD is another climate mode that results from ocean–atmosphere interaction, and causes interannual climate variability in the tropical Indian Ocean. IOD characterizes SST anomalies during this event, with warmer than normal SSTs over the western basin and cooler than usual SSTs in the eastern basin. Our study showed that decreased DMI was significantly associated with increased chikungunya incidence. A clear association is seen, with increased incidence of chikungunya when a negative DMI is observed. A positive DMI, often referred to as the positive IOD, causes drought in Indonesia, East Asia, and Australia, and flooding in some parts of the African continent [22,33]. In contrast, a negative DMI often is associated with rainfall, providing suitable conditions for *Aedes aegypti* breeding [48], potentially leading to increased chikungunya case reporting. Studies have shown that DMI is associated with fluctuations in the incidence of other infectious diseases, including malaria [23] and dengue [24,25,27].

In this preliminary study, an association between DMI and chikungunya incidence was identified. Several study limitations should be acknowledged. Notified chikungunya cases were used to represent chikungunya incidence in this study. This might be biased, as we relied solely on the report by the MoH. Since the disease has similar features to dengue, cases of chikungunya are often misdiagnosed and so lead to underreporting. Moreover, due to its challenging geographical nature, and limited access to technology, the reporting system in Indonesia is still run manually in some provinces, making it difficult for some areas to report their cases. In this study, monthly time series were used and did not include local climate variables. Therefore, a further study is needed to analyze the role of those climate variables on transmission dynamics of chikungunya using higher temporal resolutions such as weekly or daily time series data together, including lagging up to particular times, and to include other climate variables. In addition, the cross-wavelet coherence approach should be employed.

## 5. Conclusions

A substantial decline of chikungunya cases occurred in Indonesia in 2017, from the previous reporting year. This reduction did not seem to be associated with the enforcement of vector control programs by the Indonesian government. Our analysis identified an association between the climate change indicator (IOD) and chikungunya incidence in Indonesia. The present study suggests that DMI could be a potential driver of chikungunya incidence in Indonesia.
